# Evaluation of irreversible protein thermal inactivation caused by breakage of disulphide bonds using methanethiosulphonate

**DOI:** 10.1038/s41598-017-12748-y

**Published:** 2017-09-29

**Authors:** Junichiro Futami, Ai Miyamoto, Atsushi Hagimoto, Shigeyuki Suzuki, Midori Futami, Hiroko Tada

**Affiliations:** 1 Department of Medical Bioengineering, Graduate School of Natural Science and Technology, Okayama University, Okayama, 700-8530 Japan; 2 Department of Biomedical Engineering, Faculty of Engineering, Okayama University of Science, Okayama, 700-0005 Japan; 3 Division of Instrumental Analysis, Department of Instrumental Analysis and Cryogenics, Advanced Science Research Center, Okayama University, Okayama, 700-8530 Japan

## Abstract

Many extracellular globular proteins have evolved to possess disulphide bonds in their native conformations, which aids in thermodynamic stabilisation. However, disulphide bond breakage by heating leads to irreversible protein denaturation through disulphide-thiol exchange reactions. In this study, we demonstrate that methanethiosulphonate (MTS) specifically suppresses the heat-induced disulphide-thiol exchange reaction, thus improving the heat-resistance of proteins. In the presence of MTS, small globular proteins that contain disulphides can spontaneously refold from heat-denatured states, maintaining wild-type disulphide pairing. Because the disulphide-thiol exchange reaction is triggered by the generation of catalytic amounts of perthiol or thiol, rapid and specific perthiol/thiol protection by MTS reagents prevents irreversible denaturation. Combining MTS reagents with another additive that suppresses chemical modifications, glycinamide, further enhanced protein stabilisation. In the presence of these additives, reliable remnant activities were observed even after autoclaving. However, immunoglobulin G and biotin-binding protein, which are both composed of tetrameric quaternary structures, failed to refold from heat-denatured states, presumably due to chaperon requirements. Elucidation of the chemical modifications involved in irreversible thermoinactivation is useful for the development of preservation buffers with optimum constitutions for specific proteins. In addition, the impact of disulphide bond breakage on the thermoinactivation of proteins can be evaluated using MTS reagents.

## Introduction

Globular proteins usually exist in equilibrium between folded and unfolded states. Under physiological conditions, this equilibrium greatly favours the folded state. Heat-induced protein unfolding occurs near a protein’s melting temperature (T_m_), which is usually determined by differential scanning calorimetry. An unfolded protein at temperatures higher than its T_m_ displays a loss of ordered native structure, compensating for the increased polypeptide freedom. Many globular proteins remain active even after incubation at temperatures exceeding their T_m_, as they can refold into their native conformation from the heat-induced unfolded state. Thus, the reversible refolding ability from a heat-induced unfolded state is a factor in the thermal stability of a globular protein. However, even for relatively stable globular proteins, heating for long periods leads to inactivation of the protein. Detailed analysis has revealed that proteins irreversibly denatured by heat are governed by chemical modifications, including deamination of Asn/Gln residues, hydrolysis of peptide bonds at Asp-X residues, and disulphide bond scrambling^1,2^.

Industrial application of functional proteins often requires a sufficient lifetime under non-physiological conditions, or resistance to extreme conditions. Introducing extra disulphide bonds to reduce the chain entropy of unfolded states is one of the conventional approaches to achieving thermodynamic stabilisation of protein^3–11^. Conversely, disulphide bonds often enhance irreversible thermal denaturation, because free thiols generated by the destruction of disulphide bonds under heating conditions enhance disulphide bond scrambling^12^. Disulphide bond breakage and disulphide-thiol exchange reactions are accelerated under alkaline conditions^13^; therefore, these chemical modifications can be suppressed under acidic conditions. For example, recombinant insulin consists of two polypeptide chains, linked together by disulphide bonds, and is known to dissolve in an acidic buffer. Since hydrolysis of peptide bonds is accelerated under acidic and heating conditions, this acidic solvent constitution is available only at low temperatures and for proteins with spontaneous refolding abilities at physiological pH. The suppression of disulphide-thiol exchange reactions in heat-denatured proteins has been achieved through the addition of copper(II) ions^14,15^. Copper(II) ions have a high affinity for thiols and have high oxidative ability; therefore, thiols generated by heat denaturation are rapidly blocked by oxidation. However, the strong oxidative ability of copper(II) ions may cause toxicity via generation of reactive oxygen species^16^.

In order to prevent irreversible thermal denaturation of proteins, various additives have been extensively explored^1,14,17,18^. Screening has revealed that effective additives significantly suppress chemical modifications. Glycinamide is a superior additive for the prevention of irreversible denaturation^1^, through interaction with the molecular surfaces of aromatic groups, as demonstrated with hen egg lysozyme (HEL)^19^.

In this study, we investigated methanethiosulphonate (MTS) as an effective additive to specifically suppress irreversible denaturation by heat-induced disulphide-thiol exchange reactions. The small MTS molecules reacted rapidly and specifically with thiols in the heat-denatured protein, forming alkyl disulphide^20^. Because catalytic amounts of perthiol, generated by β-elimination of disulphide bonds, accelerate the disulphide-thiol exchange reaction, protection of perthiol/thiol by MTS molecules prevented irreversible denaturation. Combining MTS and other additives further decreased irreversible denaturation. The threshold for irreversible denaturation of each protein depends on the chemical and physicochemical properties of the protein. Analysis of irreversible denaturation using MTS reagents can help evaluate the contribution of disulphide bonds in the thermal stability of proteins, as well as their protein function.

## Results

### Suppression of irreversible heat-inactivation of HEL and bovine ribonuclease A (RNase A) by MTS reagents

HEL and RNase A are both small globular proteins with four intrachain disulphide bonds, and are extensively studied monomeric model proteins for irreversible denaturation. The thermodynamic stabilities of HEL and RNase A are T_m_ ~ 70 °C^21^ and T_m_ ~ 63 °C^6^, respectively. After incubation for 5 min at 100 °C, HEL was rapidly inactivated under alkaline conditions, but displayed high activity under acidic conditions (Fig. S1). As the three-dimensional structure of HEL was destroyed at any pH at 100 °C, the remaining activity indicates the rate of irreversible inactivation at heat-induced unfolded states. Although the detailed molecular mechanism is unclear, phosphate buffer accelerated the chemical reaction in the denatured protein^17^. HEPES buffer, which is a non-toxic buffer frequently used in biology, showed superior suppression of irreversible denaturation.

**Figure 1:**
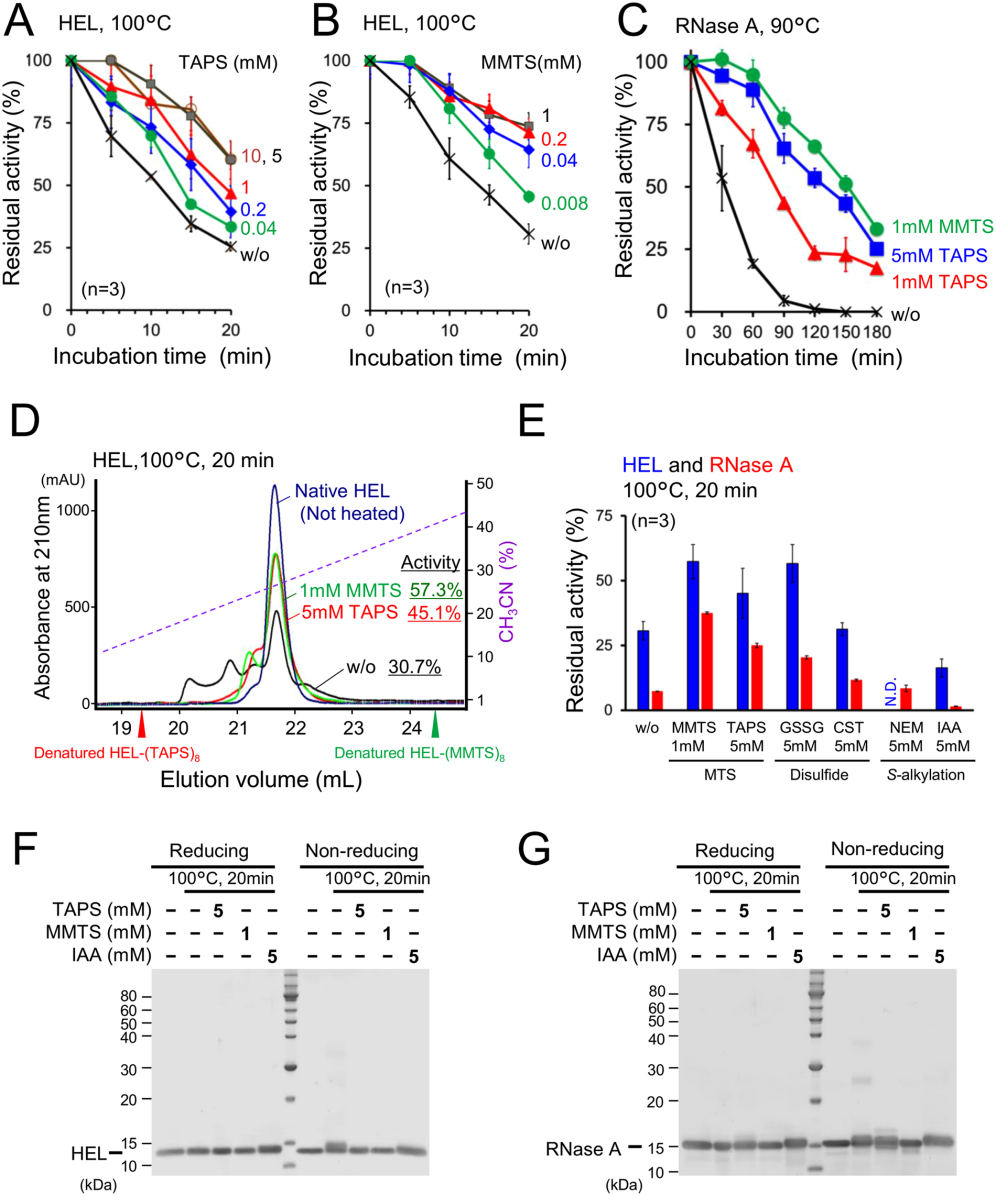
Suppression of irreversible denaturation of disulphide bond-containing small globular proteins by MTS reagents. Effects of TAPS-sulfonate (**A**) and MMTS (**B**) on the irreversible denaturation of HEL upon heating at 100 °C. (**C**) Effect of MTS reagents on the irreversible denaturation of RNase A upon heating at 90 °C. (**D**) Comparison of reversed-phase HPLC elution profiles for native and heated samples of HEL (20 µg). Elution times for denatured and fully formed alkyl disulphide derivatives of HEL are indicated by arrows. (**E**) Effects of thiol reactive reagents on irreversible denaturation of HEL and RNase A upon heating at 100 °C. N.D., not detectable. SDS-PAGE analysis of HEL (**F**) and RNase A (**G**). All heating experiments were performed in HEPES buffer, pH 6.8. Error bars indicate the standard deviation.

Both [3-(trimethylammonium)propyl] methanethiosulphonate (TAPS*-*sulfonate) and *S*-methyl methanethiosulphonate (MMTS) drastically suppressed the heat-induced irreversible denaturation of HEL and RNase A (Fig. 1A–C). The protein concentration of HEL was 0.07 mM (1 mg/mL), suggesting that an excess amount of MTS reagent is needed to suppress irreversible denaturation.

After incubation of HEL with MTS reagents at 100 °C for 20 min, residual activities were reflected by a decreased native-like peak area eluted via reversed-phase HPLC (Fig. 1D). The HEL heated with MTS reagents displayed a broader peak but distant elution time from the denatured and fully formed alkyl disulphide derivatives of HEL-(TAPS)_8_ or HEL-(MMTS)_8_. These results suggest that heated HEL possesses a limited number of thiol groups protected by MTS reagents. Indeed, mass spectrometric analysis of HEL heated with MTS reagents displayed a main fraction with a molecular mass indicating intact HEL (14305.7 Da; Fig. S2). Masses indicating possible MTS-protected fractions were barely detectable (Fig. S2).

The effects of other thiol protection reagents on heat inactivation were evaluated (Fig. 1E). The thiol-specific reversible blocker oxidised glutathione (GSSG) showed comparable effects to MTS reagents. The disulphide-containing cystamine (CST) failed to suppress protein inactivation. Furthermore, thiol-specific irreversible blockers, such as *N*-ethylmaleimide (NEM) and iodoacetamide (IAA), failed to suppress heat-induced denaturation. Taken together, these results indicate that thiol-specific rapid blocking by MTS reagents is superior at suppressing disulphide-thiol exchange reactions in disulphide-containing heat denatured globular proteins.

SDS-PAGE analysis of HEL and RNase A revealed that heat inactivation accompanied by breakage of disulphide bonds was observed by slower migration with little interchain disulphide polymerization (Fig. 1F,G).

### Analysis of the suppression mechanism for irreversible heat-inactivation of HEL by perthiol and thiol protection reagents

**Figure 2:**
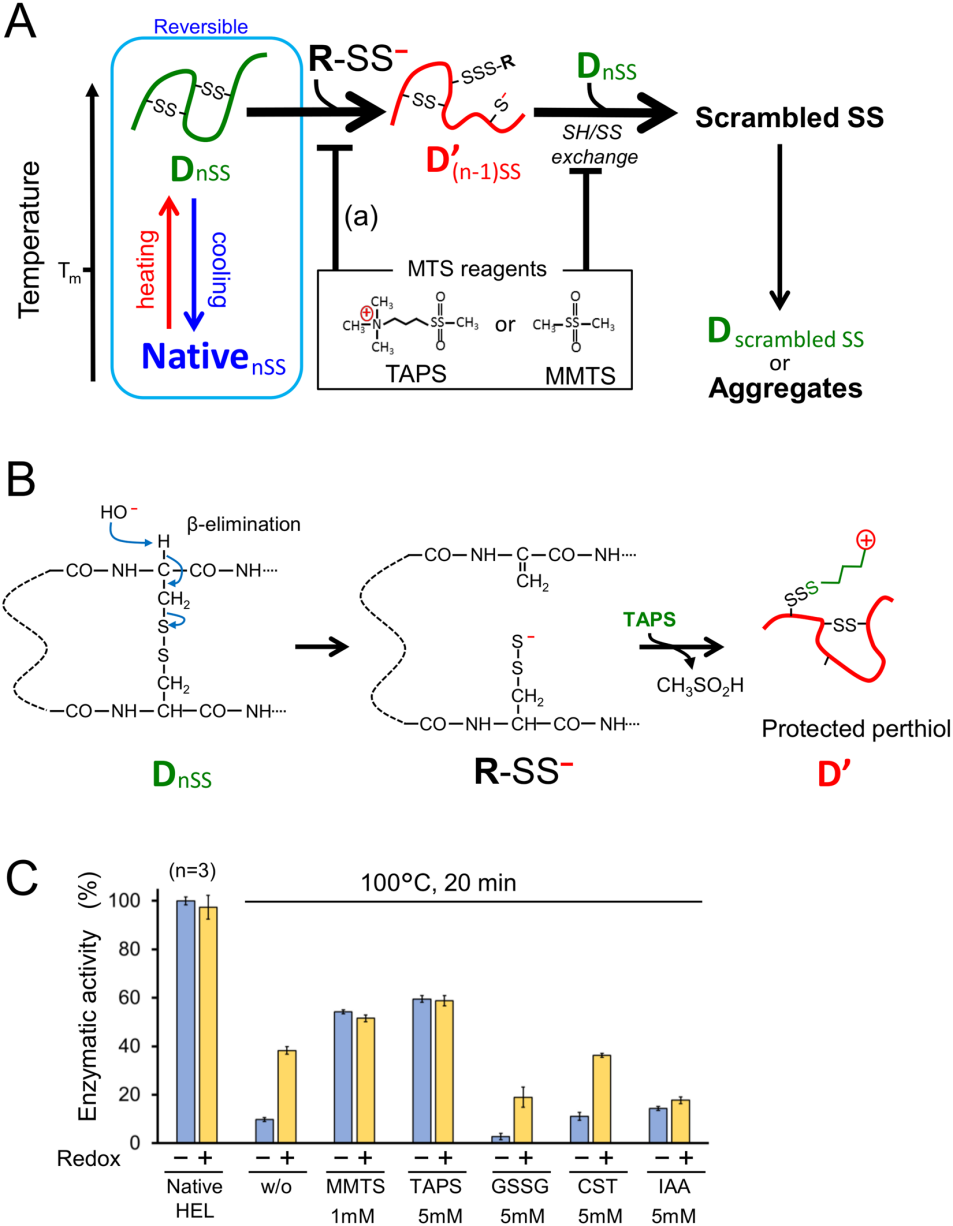
Mechanism for suppression of irreversible heat denaturation by perthiol and thiol protection reagents. (**A**) Perthiolate groups generated by the breakage of disulphide bonds induce disulphide-thiol exchange reactions upon heating, leading to irreversible denaturation by disulphide scrambling. The catalytic activity of perthiolate and thiolate can be protected rapidly and specifically by MTS reagents. (**B**) Possible mechanism for generation of perthiols by β-elimination in heat-denatured proteins. After generation of a perthiol, TAPS-Sulfonate rapidly protects the perthiolate by forming an alkyl-disulphide. (**C**) Refolding of heated HELs with additives at pH 6.8 by transferring to Tris-HCl buffer, pH 8.5 under redox conditions (2 mM GSH: 0.5 mM GSSG) or non-redox conditions for 24 h at 37 °C. Error bars indicate standard deviation.

The deduced molecular mechanisms of perthiol and thiol protection reagents to suppress irreversible denaturation during heating are summarised in Fig. 2A. In this schema, the generation of catalytic amounts of perthiolate causes irreversible inactivation of the protein by rapid disulphide-thiol exchange reactions, followed by disulphide scrambling. Although the detailed mechanism is unclear, disulphide bond breakage during heating triggered by β-elimination^1,13,14,22^ is one possible route for the generation of perthiolates (Fig. 2B). After desalting additives from heated HELs, each sample was incubated in identical redox buffers that were known to promote oxidative refolding. Enzymatic activity of HELs heated in the presence of MTS reagents were no longer changed during this redox incubation. In contrast, HELs heated with mixed disulphide reagents or without additives showed increased activity in the redox conditions (Fig. 2C). In comparison to the enzymatic activity of heated HELs before desalting (Fig. 1E), HELs heated with mixed disulphides showed decreased activity during incubation at pH 8.5. Taken together these results indicate that MTS reagents rapidly protected newly generated perthiols or thiols in the heat-denatured protein, as well as in the disulphide scrambling reaction. In contrast, HELs heated with mixed disulphide reagents leads adduct formation of disulphide scrambled products.

### Suppression of disulphhide bond breakage upon heating of bovine α-lactalbumin (BLA) by MTS reagents

**Figure 3:**
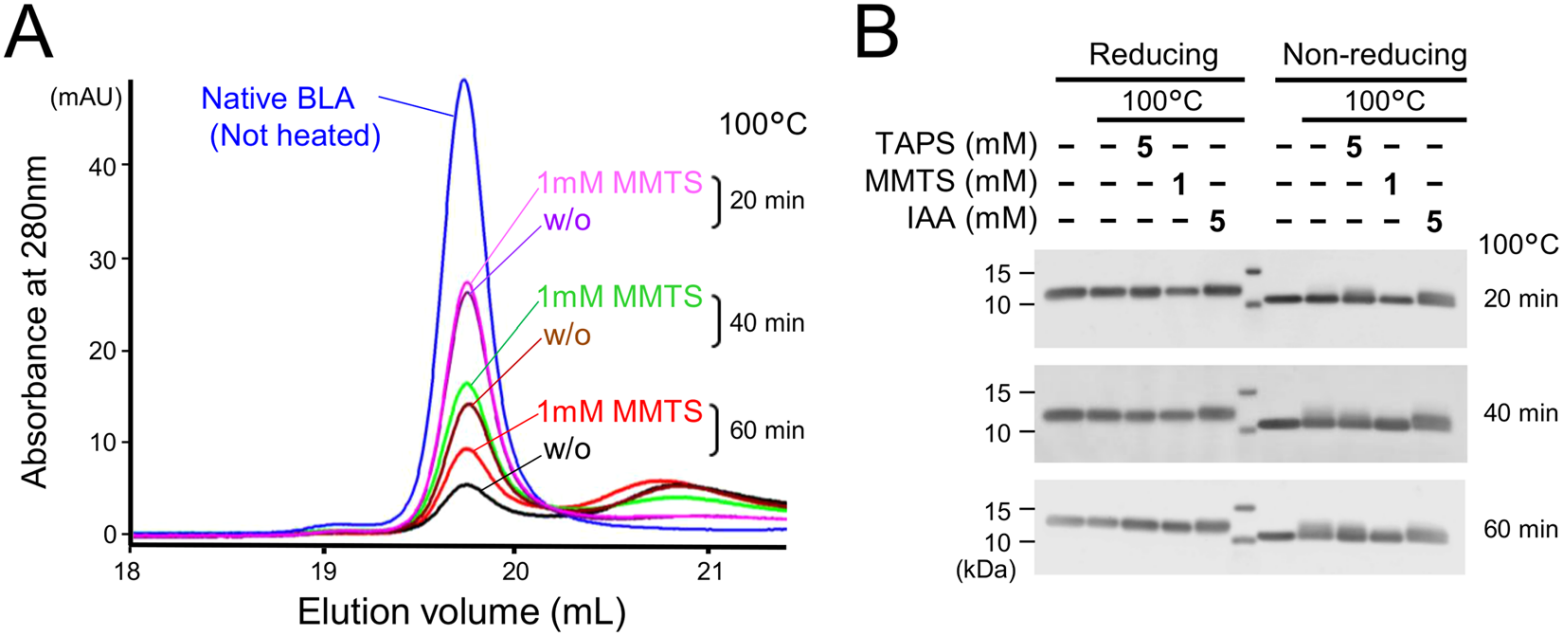
Suppression of BLA disulphide bond breakage by MMTS. (**A**) Size-exclusion HPLC elution profiles for BLA (20 µg) upon heating at 100 °C in the presence or absence of 1 mM MMTS. (**B**) SDS-PAGE analysis of BLA migration changes upon heating with or without thiol reactive reagents. All heating experiments were performed in HEPES buffer, pH 6.8.

Like HEL and RNase A, BLA has four intrachain disulphide bonds, and is an extensively studied monomeric model protein for irreversible denaturation^23^. Size-exclusion high-performance liquid chromatography (HPLC) revealed that the decrease in the monomeric BLA peak during heat incubation was partially suppressed in the presence of 1 mM MMTS (Fig. 3A). In this heating condition at pH 6.8, denatured BLA did not formed polymers but denatured, with longer retention time due to increased hydrophobicity. SDS-PAGE analysis demonstrated that heating BLA resulted in breakage of disulphide bonds, observed by slower migrating bands (Fig. 3B). These heat-induced disulphide bond breakages were drastically suppressed by MMTS.

### Effect of MTS reagents on the irreversible heat-inactivation of IgG

**Figure 4:**
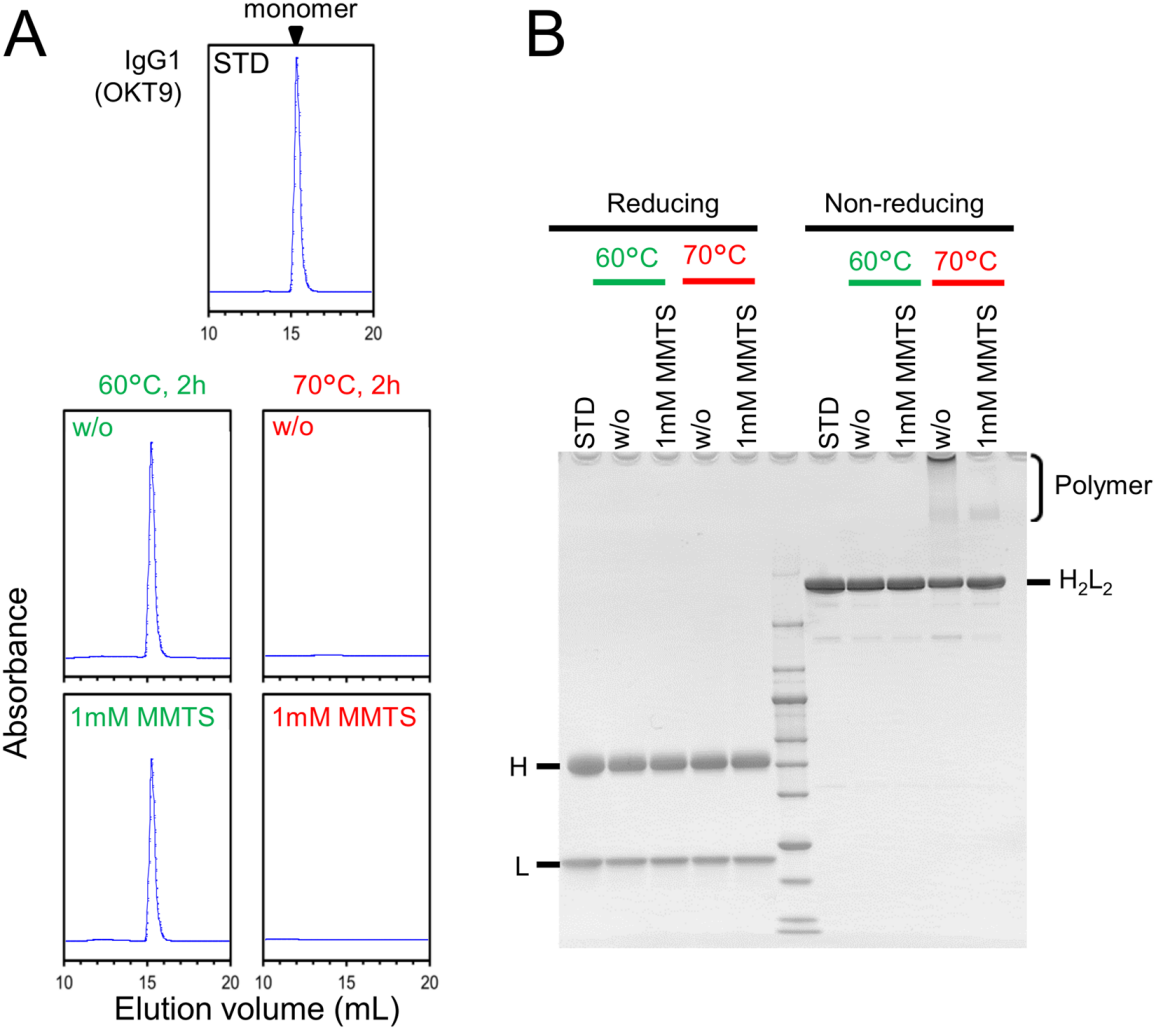
Effects of MTS reagents on IgG conformation upon heating. (**A**) Size-exclusion HPLC analysis of IgG1 (OKT9). The molecular sizes of IgG in HEPES buffer, pH 6.8, were analysed after heating at 60 °C or 70 °C for 2 h with or without 1 mM MMTS. (**B**) Disulphide-shuffled polymerization of heat-treated IgG was analysed by SDS-PAGE in the presence or absence of reducing agents.

IgG is composed of two heavy and two light chains linked together by disulphide bonds, creating a tetrameric quaternary structure. The heat-induced denaturation of IgG generally occurs in a two-step reaction comprising of a lower T_m_ (61 °C) for the F_ab_ region and a higher T_m_ (71 °C) for the F_c_ region^24^. Incubation of IgG (OKT9) at 60 °C for 2 h conserved its monomeric structure (Fig. 4) and antigen recognition ability (Fig. S3). Incubation of IgG (OKT9) at 70 °C for 2 h resulted in reduced antigen recognition ability (Fig. S3). However, monomeric IgG disappeared completely (Fig. 4A). Although MTS reagents suppressed disulphide-shuffled polymerization of IgG at 70 °C (Fig. 4B), they failed to suppress aggregation through hydrophobic interactions. These results suggest that the main cause of heat-induced denaturation of IgG is the hydrophobic interactions between the unfolded regions, as well as specific chaperon requirements for correct refolding.

### Effect of MTS reagents on engineered disulphide bonds for protein stabilisation

**Figure 5:**
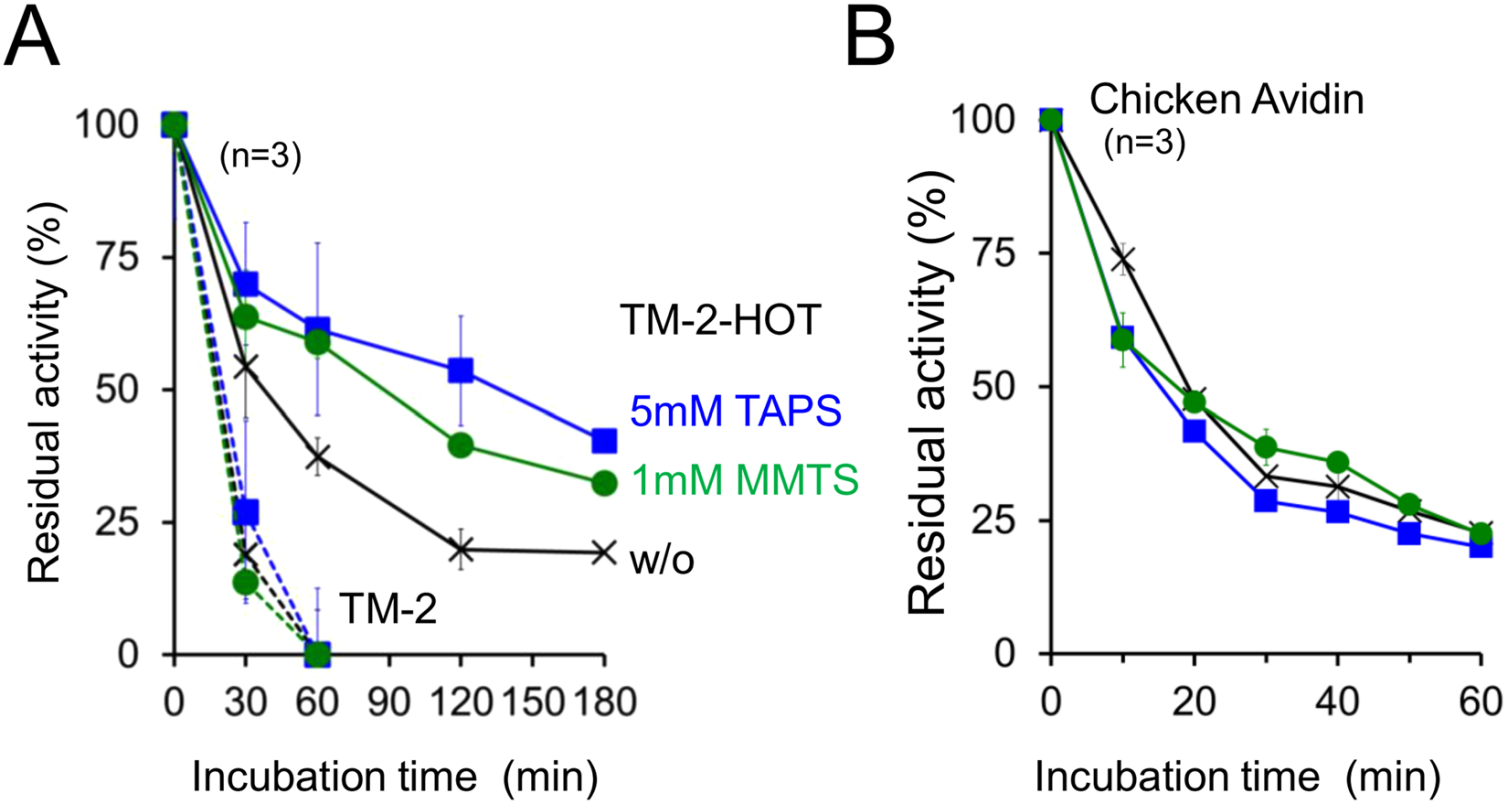
Effect of MTS reagents on the homotetrameric biotin-binding protein TM-2 upon heating. (**A**) Time-course analysis of the remaining activities of TM-2 (dashed lines) and TM-2-HOT (solid lines) after heat treatment at 100 °C in HEPES buffer, pH 6.8, with or without MTS reagents (square: TAPS, circle: MMTS). Biotin binding activity was evaluated using biotinylated-HRP after immobilisation of heat-treated protein on a microplate. (**B**) Effects of MTS reagents on chicken avidin heated at 100 °C in HEPES buffer, pH 6.8. Biotin binding activity was evaluated by HABA assay. Symbols are identical to those in panel A. Error bars indicate the standard deviation.

The biotin-binding homotetrameric recombinant protein tamavidin-2 (TM-2) shows higher thermostability (T_m_ = 85.2 °C) compared to chicken avidin (T_m_ = 78.8 °C) or streptavidin (T_m_ = 74.3 °C)^10^. TM-2-HOT, which possesses engineered intersubunit disulphide bonds, displays superior thermostability (T_m_ = 105 °C)^10^. TM-2 does not possess disulphide bonds; therefore, MTS reagents did not affect it (Fig. 5A). Chicken avidin, which possesses one disulphide bond pair in each subunit, but no cross linking between intersubunits, was denatured even in presence of MTS reagents (Fig. 5B), suggesting that unfolded chicken avidin dissociates to monomers. However, TM-2-HOT was successfully stabilised by MTS reagents (Fig. 5A), suggesting that suppression of intersubunit disulphide bond breakage enabled refolding from heat-denatured states.

### Enhanced stabilisation of protein at extreme conditions

**Figure 6:**
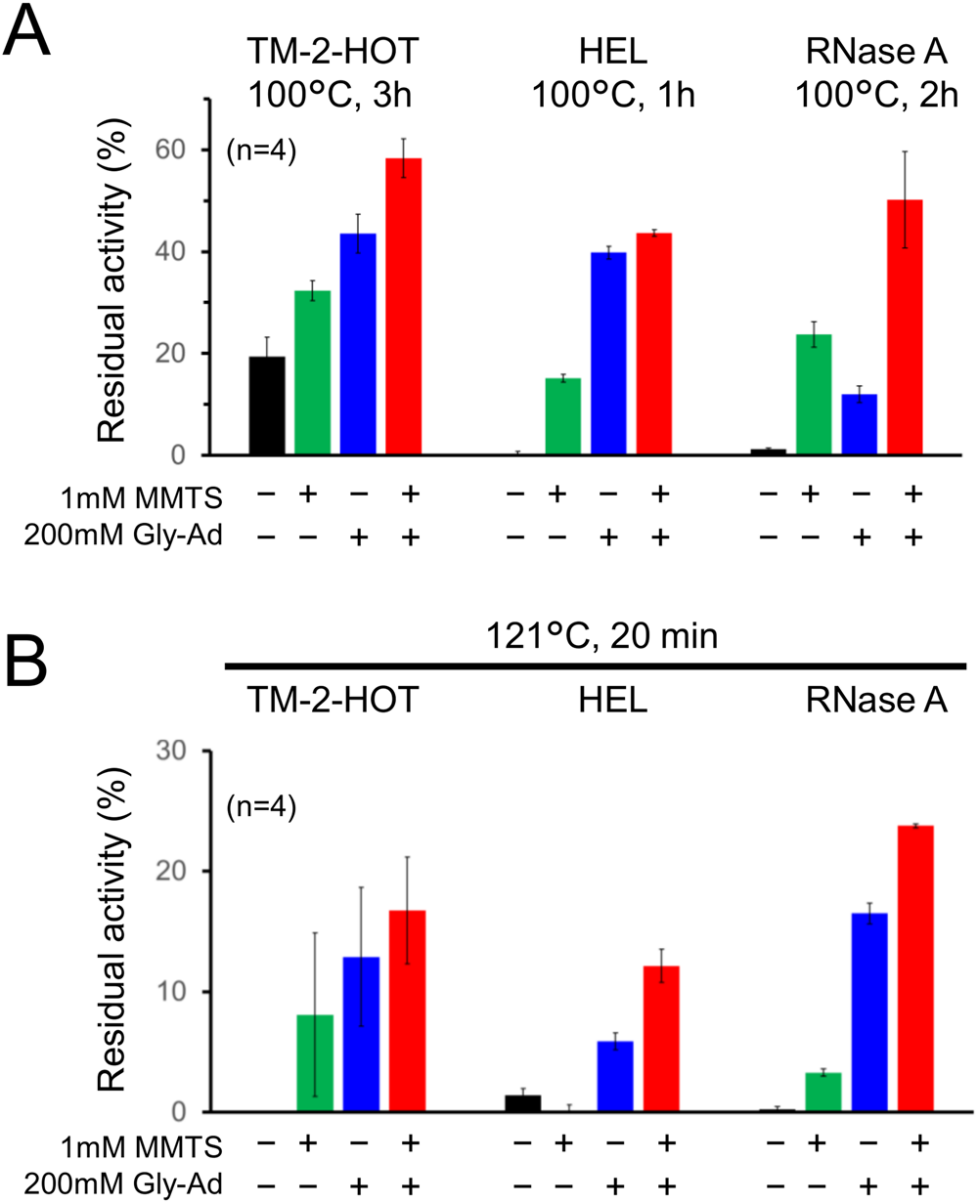
Enhanced suppression of irreversible denaturation of protein by combining glycineamide and MTS reagents. (**A**) Remaining activity of TM-2-HOT, HEL, and RNase A heated at 100 °C in HEPES buffer, pH 6.8, with defined additives. (**B**) Survival of functional TM-2-HOT, HEL, and RNase A after autoclaving with additives. Error bars indicate the standard deviation.

Several additives are effective in suppressing irreversible protein denaturation. In this study, we evaluated the enhanced stabilisation effect of MTS reagents in combination with the effective additive glycinamide^1^. As shown in Fig. 6A, TM2-HOT, HEL, and RNase A showed enhanced stabilisation upon heating at 100 °C with the addition of 1 mM MMTS and 200 mM glycinamide. The enhanced stabilisation effects were confirmed even after autoclaving (Fig. 6B). Despite extensive chemical modifications, the molecular mass of HEL (14305.7 Da) remained intact after autoclaving, as detected by mass spectrometry (Fig. S4).

## Discussion

The properties of mammalian intracellular and extracellular proteins have evolutionarily adapted to their opposing environments. The disulphide-free, flexible conformation of intracellular proteins allows multiple protein interactions in living cells under highly crowded conditions. This structural property is suggested to contribute to the enhanced solubility of intracellular proteins^25^. Conversely, extracellular proteins need to be robust to circulate in the extracellular space. Disulphide bonds substantially contribute to the stabilisation of proteins by decreasing the chain entropy of the unfolded state^26^. Although refolding from reduced and denatured states requires appropriate redox conditions^27^, the refolding of small globular proteins is a virtually spontaneous process, provided that intrachain disulphide bonds are intact. Therefore, conserving wild-type disulphide bonds is critical for the suppression of irreversible denaturation during heating conditions (Fig. 2A).

The generation of thiols from a disulphide-containing protein with heating is an unavoidable process at physiological pH^22^. The most likely candidate for this process is β-elimination of disulphide bonds, which readily takes place upon heating in alkaline conditions. Although β-elimination is not expected to be highly prevalent at pH 6.8^13,14^, newly generated perthiols must be rapidly protected for suppression of disulphide-thiol exchange reactions and disulphide scrambling (Fig. 2A,B). It is important to note that perthiol of cysteine is more reactive at pH 6.8, because the pKa value for perthiol is 1 to 2 pKa units more acidic than that for thiol^28^. As shown in Figs 1 and 2, MTS-reagents are superior additives to supress the disulphide scrambling reactions in heat-denatured proteins. However, MTS-protected fractions were barely detectable by mass spectrometric analysis (Fig. S2). These results suggest that extremely small amount of generated perthiols in heat-denatured proteins trigger an irreversible denaturation by disulphide scrambling.

MTS reagents are a reasonable choice to suppress irreversible denaturation of globular proteins triggered by disulphide bond breakage due to their high thiol-specificity and rapid protection ability. Traditionally, copper(II) ions were used as an effective suppressor of disulphide-thiol exchange protein denaturation^14,15^, but copper(II) catalyses unfavourable methionine oxidation, and the formation of copper(II) hydroxide precipitates often interferes with optical analysis^29^. Thiol-specific irreversible blockers, such as NEM and IAA, protect thiols generated by breakage of disulphide bonds upon heating by *S*-alkylation. However, neither NEM nor IAA suppressed the heat-induced irreversible denaturation of HEL and RNase A, presumably due to side-reactions (Fig. 1E). Both NEM and IAA had side-reactions with other amino acid residues upon heating. For instance, heated HEL with NEM displayed aggregation, possibly due to reactions with amine groups, and heated RNase A with IAA was inactivated by modification of active site His residues. Although mixed disulphide reagents like GSSG displayed a higher suppression of heat-induced HEL denaturation (Fig. 1E), the products appeared to contain disulphide-scrambled proteins (Fig. 2C). Because mixed disulphide reagents react rapidly to perthiols or thiols but do not abolish them in the system, the remaining perthiols or thiols enhance the scrambling reaction to produce enzymatically reactivatable disulphide scrambled products.

Unlike mixed disulphides, MTS reagents form sulphinic acid as a byproduct of the reaction, decomposing into a volatile product without affecting the disulphide bonds^20^. Based on this molecular mechanism, dipyridyl disulphide reagents could be an alternative suppressor. However, low solubility in aqueous buffer limits their application. Positively charged TAPS-sulfonate is a highly water-soluble MTS reagent, soluble at concentrations of more than 2 M^30^, whereas MMTS is soluble up to 1 mM in aqueous buffer. Various protein engineering applications have been developed based on the highly water-solubility of *S*-cationised unfolded proteins^31–34^. TAPS-sulfonate was predicted to have higher efficiency, due to improved solubility of the transient SH-protected unfolded protein intermediate. However, in this study, 1 mM MMTS was superior; this suggests that the relatively lower steric hindrance of MMTS may be more favourable for rapid reaction with produced thiols than enhanced solubility.

There are typically two types of globular proteins: some are able to spontaneously fold from its unfolded state to a thermodynamically stable state, while others require external energy from chaperons to achieve correct folding. Both RNase A and HEL are extensively studied, spontaneously foldable proteins. Thus, the unfolded proteins, possessing wild-type pairs of disulphide bonds, rapidly folded to their stable native conformations (Figs 1–3). The folding of IgG *in vivo* requires assembly of partner domains, which is associated with the endoplasmic reticulum chaperon, BiP^35^. Although the MTS reagents suppressed disulphide shuffling of IgG under heating conditions, irreversible intermolecular aggregation of misfolded proteins occurred preferentially (Figs 4 and S3). Dissociation of subunits from homotetramic chicken avidin and TM-2 appear to be main routes for their irreversible denaturation (Fig. 5), while the suppression of intersubunit disulphide bond breakage in TM-2-HOT diminished irreversible denaturation. Analysis of the effects of MTS reagents on heat-induced irreversible denaturation facilitates understanding the weak points of specific proteins with regards to folding.

Higher protein heat stability could be advantageous for industrial use, and proper selection of additives to suppress irreversible denaturation can improve the shelf life of proteins. There are a number of previous studies regarding the ability of additives to suppress chemical modifications, but there are currently no reports regarding the application of MTS reagents for the suppression of disulphide shuffling. Here, we have successfully demonstrated that MTS reagents can suppress irreversible denaturation of proteins upon heating (Fig. 6).

Sterilisation of biologics and medical equipment is required under legally defined conditions. Heat-sensitive proteins are usually sterilised by filtration to remove bacterial contaminants, as protein refolding after autoclaving is rarely observed. In this study, we demonstrate the possibility of autoclave sterilisation of proteins (Fig. 6B). Autoclaving is known to insufficient at eliminating RNase from solutions, addition of diethyl pyrocarbonate to modify catalytic histidine residues is recommended for RNA-based molecular biological procedures. The combining appropriate additives to suppress chemical modifications of protein under autoclaving conditions may pave the way for reliable sterilisation of medical equipment combined with functional proteins.

## Methods

### Materials

HEL (uniprot:P00698), RNase A (bovine pancreatic Type XII-A, uniprot:P61823), BLA (bovine Type I, uniprot: P00711), *Micrococcus lysodeikticus* (ATCC No. 4698), yeast tRNA (Type X), N-2-Hydroxyethylpiperazine-N′-2-ethane sulfonic acid (HEPES), cystamine dihydrochloride (CST), and MMTS were purchased from Sigma-Aldrich (St. Louis, MO, USA). Chicken avidin (uniprot: P02701), reduced glutathione (GSH) and oxidized glutathione (GSSG) were purchased from Nacalai Tesque (Kyoto, Japan). TAPS*-*sulfonate was purchased from Katayama Chemical (Osaka, Japan). Glycinamide, NEM, and IAA were purchased from Wako Chemical (Osaka, Japan).

### Preparation of recombinant proteins

Expression and purification of recombinant TM-2 (uniprot: B9A0T7) and TM-2-HOT^10^ was performed as previously described^36^. Monoclonal anti-human transferrin receptor antibody (OKT9, IgG1) was produced by cultivating the hybridoma (CRL-8021, ATCC, Manassas, VA, USA) in CD Hybridoma medium (Life Technologies, Carlsbad, CA, USA) with orbital shaking at 37 °C in the presence of 8% CO_2_ for 4 days. Secreted OKT9-IgG protein was purified using a HiTrap Protein G HP column (GE Healthcare, Piscataway, NJ, USA), according to the manufacturer’s instructions.

### Heating conditions

Each protein was adjusted to 1 mg/mL in 50 mM HEPES or phosphate buffer with defined additives. The pH value after addition of all additives was adjusted using a pH meter (F-52, HORIBA, Kyoto, Japan). Protein solutions (100 µL), which were tightly sealed in 0.2-mL PCR tubes, were incubated in a block-incubator (BI-516H, ASTEK, Fukuoka, Japan) or autoclave (121 °C for 20 min, SX-500, TOMY, Tokyo, Japan). Heat-treated protein solutions were immediately cooled on ice, and stored at 4 °C until use.

### Analysis of heated HEL by reversed-phase HPLC

HEL (1 mg/mL in 50 mM HEPES buffer, pH 6.8) was left in native form or heated at 100 °C for 20 min with or without MTS reagents (1 mM MMTS or 5 mM TAPS*-*sulfonate), and analysed using a reversed-phase HPLC column (YMC-Pack ODS-A, 6.0 mm I.D. × 150 mm, YMC, Kyoto, Japan) by an acetonitrile linear gradient elution procedure in the presence of 0.1% HCl. Denatured and fully formed *S*-alkyl disulphide conjugates of HEL-(TAPS)_8_ and HEL-(MMTS)_8_ were prepared as previously described^31^.

### Refolding assay of heat-denatured HEL

After heating 8 × 100 µL of HEL solutions (1 mg/mL in 50 mM HEPES buffer, pH 6.8) with defined additives at 100 °C for 20 min, each solvent was buffer exchanged to 50 mM Tris-HCl buffer, pH 8.5, by using a PD-10 column (GE Healthcare). Samples were then diluted to 0.1 mg/mL final protein concentration in 50 mM Tris-HCl buffer, pH 8.5, with or without 2 mM GSH and 0.5 mM GSSG. After incubation of the refolding samples at 37 °C for 24 h, samples were stored at 4 °C until used in enzymatic assays.

### Analysis of residual activity

The enzymatic activity of HEL against *M*. *lysodeikticus* was determined according to a turbidimetric method at 450 nm in phosphate buffered saline at pH 7.4^37^. RNase activity toward yeast tRNA was assayed by determining the generation of acid-soluble digested RNA^38^. The thermal stability of TM-2 and TM-2-HOT was assayed using a microplate-scale assay. Heat-treated proteins were immobilised on a microtitre plate, and the biotin-binding activity was assessed using biotinylated horseradish peroxidase^10^. The biotin-binding activity of chicken avidin was determined by changes in absorbance at 500 nm after mixing with 2-(4-hydroxyazobenzene)benzoic acid (HABA).

### Analysis of molecular size by size-exclusion HPLC and SDS-PAGE

The residual monomeric BLA, IgG, and their soluble aggregates were evaluated by size exclusion chromatography (COSMOSIL 5Diol-300-II, 7.5 mm I.D. × 600 mm, Nacalai Tesque), equilibrated with 50 mM sodium phosphate buffer, pH 6.5, at a flow rate of 0.5 mL/min. Heat-induced disulphide-scrambled and polymerised products were analysed by SDS-PAGE. Reducing samples were prepared by incubation at 80 °C for 5 min in 1 × sample buffer solution with reducing reagent, whereas non-reducing samples were prepared in 1 × sample buffer without reducing reagent (Nacalai Tesque), and were not heated. Gels were stained with Coomassie Brilliant Blue.

### Data availability

No datasets were generated during the current study.

## Electronic supplementary material

Supplementary Information
