# Supplementary material for: Evaluation of irreversible protein thermal inactivation caused by breakage of disulphide bonds using methanethiosulphonate
Source: Sci Rep. 2017 Sep 29;7:12471. doi: 10.1038/s41598-017-12748-y (PMC5622167; doi:10.1038/s41598-017-12748-y)
Supplement: Supplementary file 1 — Supplementary Information [file 41598_2017_12748_MOESM1_ESM.pdf]

Supplementary Information

Evaluation of irreversible protein thermal inactivation caused by breakage of disulphide bonds using methanethiosulphonate

Junichiro Futami[1*], Ai Miyamoto[1], Atsushi Hagimoto[1], Shigeyuki Suzuki[1], Midori Futami[2] and Hiroko Tada[3]

[1]Department of Medical Bioengineering, Graduate School of Natural Science and Technology, Okayama University, Okayama 700-8530, Japan
[2]Department of Biomedical Engineering, Faculty of Engineering, Okayama University of Science, Okayama, 700-0005, Japan
[3]Division of Instrumental Analysis, Department of Instrumental Analysis and Cryogenics, Advanced Science Research Center, Okayama University, Okayama 700-8530, Japan

***Corresponding author**
Junichiro Futami, Ph.D.
Department of Medical Bioengineering, Graduate School of Natural Science and Technology, Okayama University, Okayama 700-8530, Japan
Phone & Fax: 81-86-251-8217
E-mail: futamij@okayama-u.ac.jp

Supplementary information consists of Figure S1 to S4

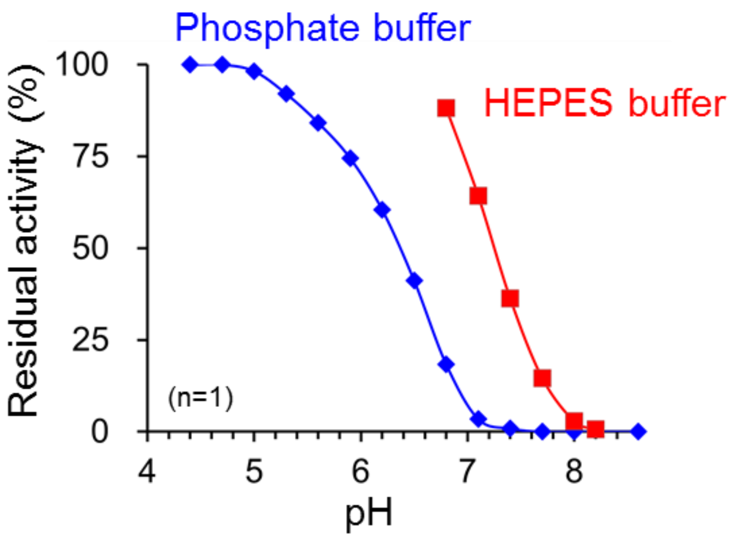

Figure S1. pH-dependent thermoinactivation of HEL. HEL solubilized (1 mg/mL) in 50 mM of HEPES or phosphate buffer at various pH values were treated at 100 °C for 5 min, and then assayed for residual enzymatic activity against *Micrococcus lysodeikticus*.

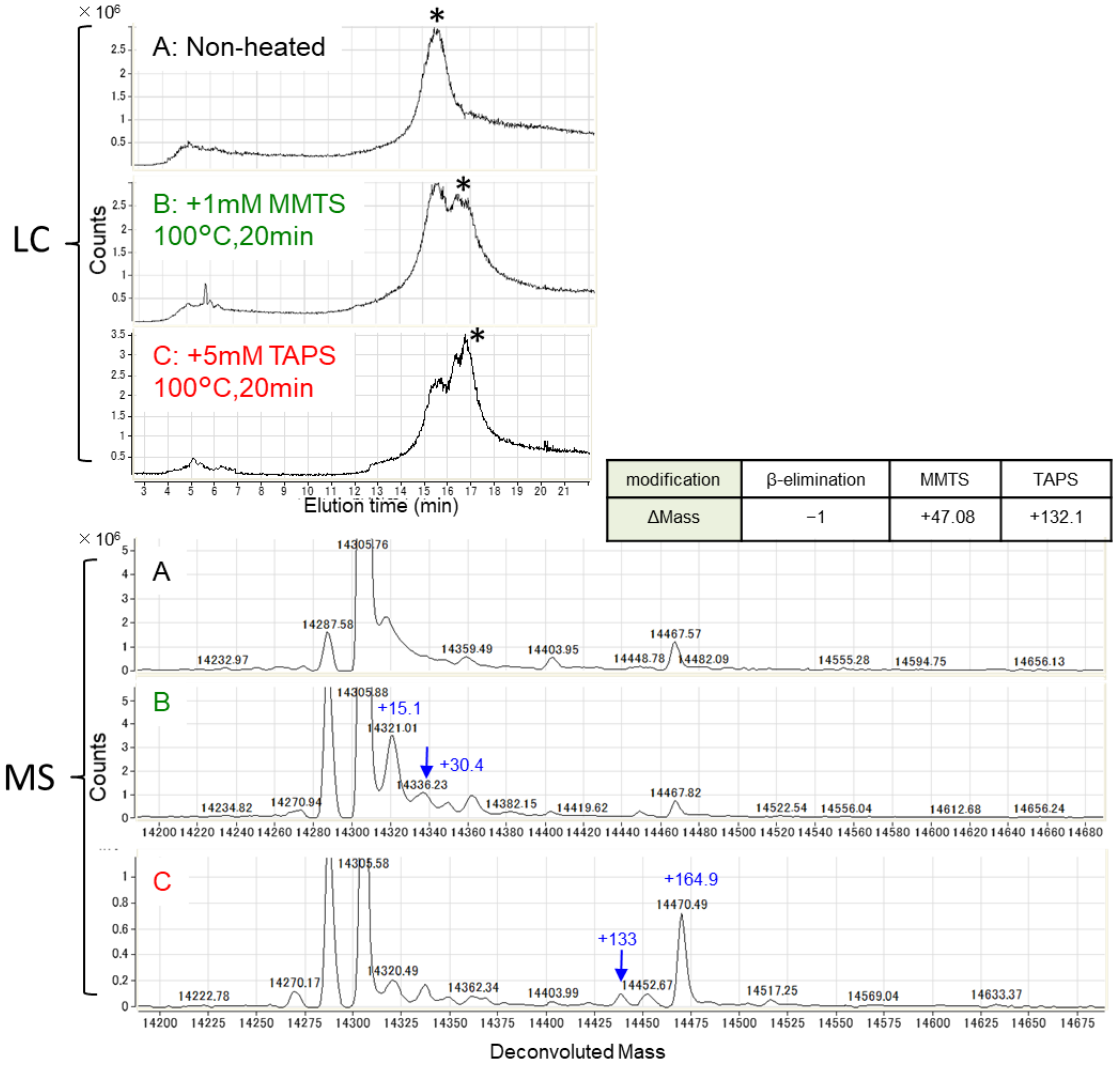

modification	β-elimination	MMTS	TAPS
ΔMass	−1	+47.08	+132.1

Figure S2. Mass spectrometric analysis of HEL after heating in the presence of MTS reagents. Deconvoluted mass spectra of non-heated HEL and heated HELs (1 mg/mL) in 50 mM HEPES buffer, pH6.8 in the presence of 1 mM MMTS (B) or 5mM TAPS-Sulfonate. Molecular mass of HEL containing in marked (*) peak on protein-chip LC were analyzed by HPLC-CHIP/QTOF mass spectrometry (G6520 Agilent Technologies, CA, USA). Every samples showed high intensity as the mass for native HEL.

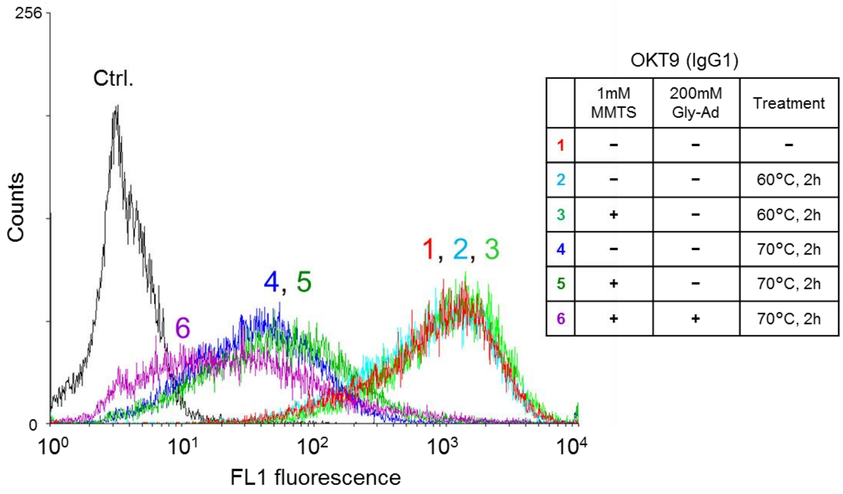

Figure S3. Evaluation of antigen recognition activity of OKT9. Monoclonal antibodies (mouse IgG1) against human transferrin receptor (OKT9, 1 mg/mL) with various additives were heated under the indicated conditions. After dilution of each sample to a concentration of 20 µg/mL in PBS containing 0.1% BSA, samples were incubated with trypsinized HeLa cells for 1 h. After washing twice with PBS, cells were incubated with Alexa488-conjugated anti-mouse IgG, and used for flow cytometry analysis (Cell Lab Quanta SC, Beckman Coulter, CA, USA).

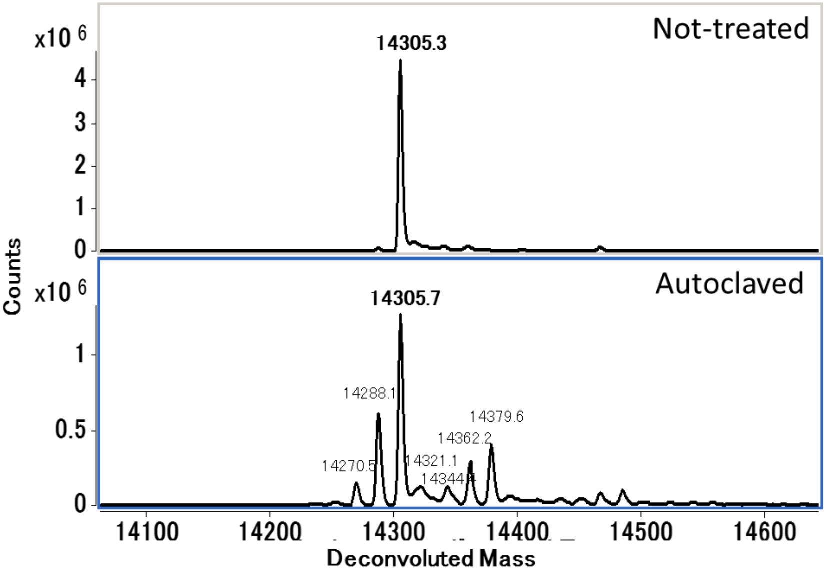

Figure S4. Mass spectrometric analysis of HEL after autoclaving. Deconvoluted mass spectra of non-treated HEL and autoclaved HEL in 50 mM HEPES buffer consisting of 200 mM Gly-Ad and 1 mM MMTS. Molecular mass of each sample was analyzed by HPLC-CHIP/QTOF mass spectrometry (G6520 Agilent Technologies, CA, USA).
